# Phospholamban inhibits the cardiac calcium pump by interrupting an allosteric activation pathway

**DOI:** 10.1016/j.jbc.2024.107267

**Published:** 2024-04-06

**Authors:** Sean R. Cleary, Jaroslava Seflova, Ellen E. Cho, Konark Bisht, Himanshu Khandelia, L. Michel Espinoza-Fonseca, Seth L. Robia

**Affiliations:** 1 Department of Cell and Molecular Physiology, Loyola University Chicago, Maywood, Illinois, USA; 2 Department of Physics, Chemistry, and Pharmacy, PHYLIFE: Physical Life Science, University of Southern Denmark, Odense, Denmark; 3 Division of Cardiovascular Medicine, Department of Internal Medicine, Center for Arrhythmia Research, University of Michigan, Ann Arbor, Michigan, USA

**Keywords:** allosteric regulation, biosensor, calcium ATPase, calcium transport, cardiac muscle, fluorescence resonance energy transfer (FRET), molecular dynamics simulations

## Abstract

Phospholamban (PLB) is a transmembrane micropeptide that regulates the sarcoplasmic reticulum Ca^2+^-ATPase (SERCA) in cardiac muscle, but the physical mechanism of this regulation remains poorly understood. PLB reduces the Ca^2+^ sensitivity of active SERCA, increasing the Ca^2+^ concentration required for pump cycling. However, PLB does not decrease Ca^2+^ binding to SERCA when ATP is absent, suggesting PLB does not inhibit SERCA Ca^2+^ affinity. The prevailing explanation for these seemingly conflicting results is that PLB slows transitions in the SERCA enzymatic cycle associated with Ca^2+^ binding, altering transport Ca^2+^ dependence without actually affecting the equilibrium binding affinity of the Ca^2+^-coordinating sites. Here, we consider another hypothesis, that measurements of Ca^2+^ binding in the absence of ATP overlook important allosteric effects of nucleotide binding that increase SERCA Ca^2+^ binding affinity. We speculated that PLB inhibits SERCA by reversing this allostery. To test this, we used a fluorescent SERCA biosensor to quantify the Ca^2+^ affinity of non-cycling SERCA in the presence and absence of a non-hydrolyzable ATP-analog, AMPPCP. Nucleotide activation increased SERCA Ca^2+^ affinity, and this effect was reversed by co-expression of PLB. Interestingly, PLB had no effect on Ca^2+^ affinity in the absence of nucleotide. These results reconcile the previous conflicting observations from ATPase assays *versus* Ca^2+^ binding assays. Moreover, structural analysis of SERCA revealed a novel allosteric pathway connecting the ATP- and Ca^2+^-binding sites. We propose this pathway is disrupted by PLB binding. Thus, PLB reduces the equilibrium Ca^2+^ affinity of SERCA by interrupting allosteric activation of the pump by ATP.

The sarcoplasmic reticulum Ca^2+^-ATPase (SERCA) sequesters intracellular Ca^2+^ into the lumen of the endoplasmic reticulum (ER) to establish a reservoir for cell signaling. This is a critically important process in all cell types and is energized by ATP hydrolysis and catalytic autophosphorylation of the Ca^2+^ pump. Ca^2+^ transport plays a particularly central role in cardiac physiology. The release of Ca^2+^ from the sarcoplasmic reticulum (SR) initiates the shortening of the cardiac muscle cell during systole (cardiac contraction). Then, SERCA transport removes Ca^2+^ from the cytosol during diastole (cardiac relaxation) and re-establishes the Ca^2+^ stores in preparation for the next cardiac cycle (1, 2). The primary regulator of SERCA function in the heart is phospholamban (PLB), a transmembrane micropeptide that physically interacts with SERCA and inhibits Ca^2+^ transport (3, 4). PLB regulation of SERCA is known to be critical for human survival since naturally occurring mutations of PLB that nullify its inhibition are associated with heart failure and premature death by the third decade in carriers (5). PLB reduces the Ca^2+^ sensitivity of SERCA during cycling, increasing the Ca^2+^ concentration required for pump turnover (6, 7). However, equilibrium measurements of Ca^2+^ binding (in the absence of ATP) have not shown any effect of PLB on the affinity of SERCA for Ca^2+^ (6, 8, 9). These apparently contradictory results have been reconciled by invoking a kinetic mechanism, that PLB slows structural transitions of the transporter associated with the Ca^2+^ binding step of the SERCA enzymatic cycle. This could account for the observed Ca^2+^ desensitization effect of PLB without actually reducing the Ca^2+^ affinity of SERCA under equilibrium conditions (6).

Alternatively, we speculated that experiments measuring Ca^2+^ binding in the absence of ATP may overlook important allosteric effects of bound nucleotides. In addition to the role of ATP as a source of energy to fuel Ca^2+^ transport, ATP binding to SERCA increases the transporter's affinity for Ca^2+^. This effect of ATP, referred to as “nucleotide activation”, increases both the rate of Ca^2+^ binding (10, 11, 12) and the Ca^2+^ affinity of the pump measured using ^45^Ca^2+^ (13, 14). Since the effects of nucleotide activation are the opposite of PLB inhibition, we hypothesize that PLB may inhibit SERCA through a mechanism of reversing nucleotide activation, thereby actually reducing the equilibrium binding affinity of SERCA for Ca^2+^. Since previous experiments measuring the impact of PLB on Ca^2+^-binding were always performed in the absence of ATP (to prevent enzymatic cycling) (6, 8, 9), to our knowledge this possibility has not been investigated. To test this mechanistic hypothesis, we investigated the interplay of ATP binding, Ca^2+^ binding, and PLB binding using a biosensor that reports SERCA conformation through changes in intramolecular fluorescence resonance energy transfer (FRET) (15). This biosensor-based assay offers significantly improved sensitivity compared to conventional ^45^Ca^2+^-binding measurements. The results support a new paradigm for the mechanism of regulation of SERCA by PLB.

## Results

### PLB reverses nucleotide activation of SERCA Ca^2+^ affinity by ATP

**Figure 1:**
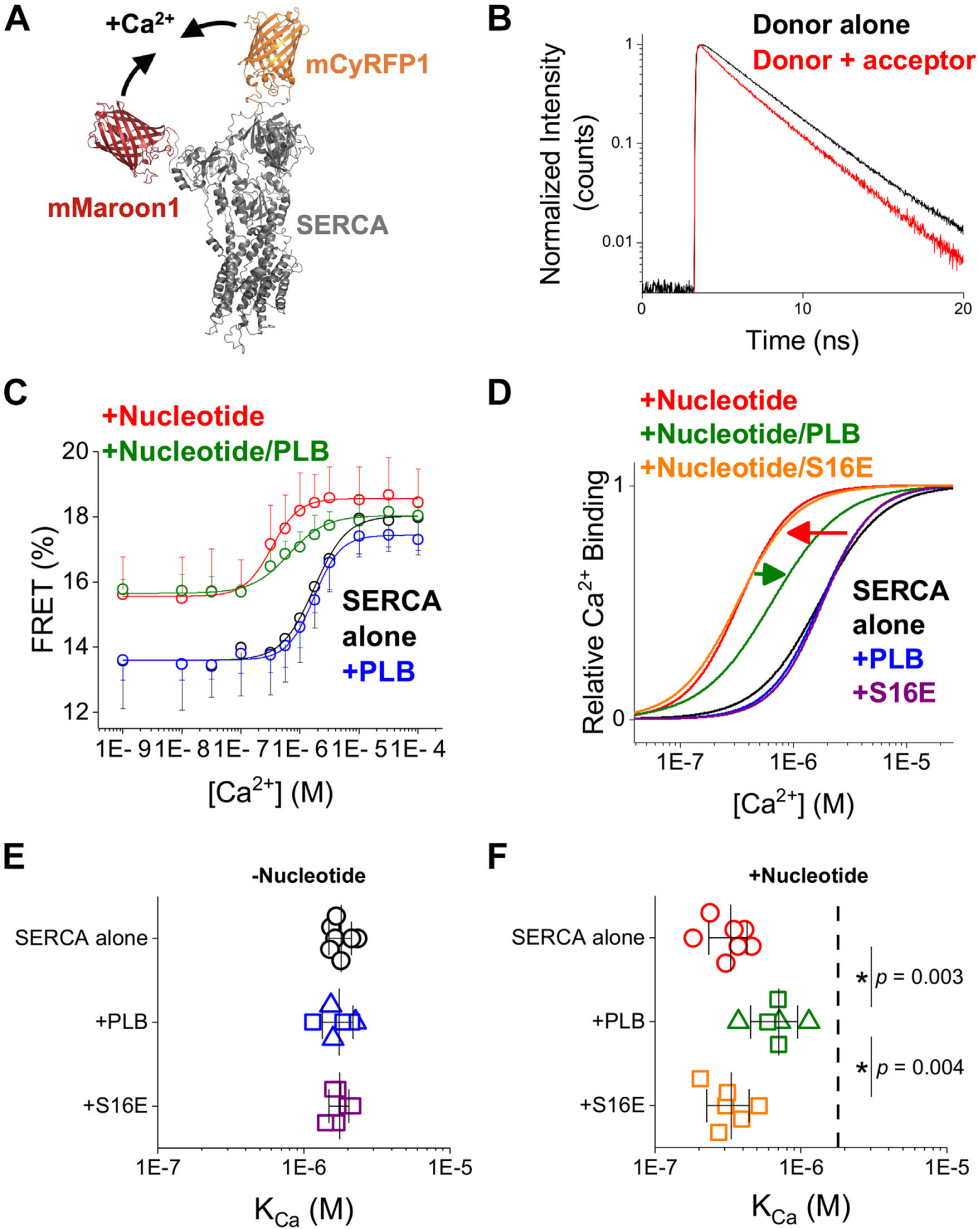
**PLB reduces the Ca**^**2+**^**affinity of SERCA by reversing nucleotideactivation.***A*, 2-color SERCA biosensor labeled on the A and N domains detects headpiece closure during Ca^2+^ binding by intramolecular FRET. *B*, biosensor FRET changes were quantified from donor fluorescence lifetime. *C*, FRET increased with increased Ca^2+^, shown for SERCA alone (*black*), SERCA+nucleotide (*red*), SERCA co-expressed with PLB (*blue*), and SERCA+ nucleotide+PLB (*green*). *D*, fits to the data in C, normalized to show changes in Ca^2+^ affinity. Normalized data for samples co-expressing PLB-S16E are shown with (*orange*) and without (*purple*) nucleotide (See Fig. S3). *E*, apparent K_Ca_ of SERCA alone (*black*), or with co-expression of WT- (*blue*) or S16E-PLB (*purple*) in the absence of nucleotide. *F*, apparent K_Ca_ of SERCA alone (*red*) with co-expression of WT- (*green*) or S16E-PLB (*orange*) in the presence of nucleotide. SERCA to PLB transfection ratio is indicated by Δ (1:3) or □ (1:5). Lines and error bars represent mean ± SD. Differences in K_Ca_ were evaluated by one-way ANOVA for the -nucleotide (*F* = 0.05, *p* = 0.952) and +nucleotide (*F* = 10.63, *p* = 0.001) conditions with Dunn-Sidak post hoc (∗*p* < 0.05, SERCA alone *n* = 7, +PLB *n* = 6, +S16E *n* = 6).

We previously developed a biosensor called “2-color SERCA” (Fig. 1*A*) consisting of two fluorescent proteins fused to the A- and N- domains of the cytoplasmic headpiece of SERCA to report its overall conformation by intramolecular FRET (9, 15, 16, 17). Here, we used this biosensor to study the activating and inhibitory effects of ATP and PLB, respectively, on SERCA Ca^2+^ affinity. FRET was measured from the mCyRFP1 donor to the mMaroon1 acceptor for 2-color SERCA expressed in microsomal fractions from HEK-293T cells. Specifically, we used time-correlated single photon counting (TCSPC) to quantify FRET by measuring the decrease in the mCyRFP1 donor fluorescence lifetime in close proximity to the acceptor (Figure 1*B*, S1, See Experimental Procedures). 2-color SERCA FRET increased in response to increasing Ca^2+^ concentration (15) (Fig. 1*C*, *black*). The data were well-described with a Hill function with minimal FRET efficiency at low [Ca^2+^] of 13.6 ± 1.3%, a maximum of 18.0 ± 1.0% at high [Ca^2+^], and a Ca^2+^ binding constant (K_Ca_) of 1.8 ± 0.3 μM for SERCA alone (*mean ± SD*). This value is consistent with previous measurements of 2-color SERCA Ca^2+^ affinity (9, 15, 16). The addition of the non-hydrolyzable ATP analog, AMPPCP (500 μM) shifted the Ca^2+^ binding curve to the left, indicating an increase in Ca^2+^ affinity (K_Ca_ of 332 ± 97 nM, *t* test *p* = 6.1E-8) (Fig. 1*C*, *red*). We noted that FRET at low [Ca^2+^] was significantly increased when nucleotide was present to 15.6 ± 1.2% (*t* test *p* = 0.01, Fig. S2), consistent with a more compact SERCA headpiece after nucleotide binding (16, 18). For ease of comparison of Ca^2+^ affinity of SERCA under different conditions, the fits of the data in Figure 1*C* are shown again in Figure 1*D* normalized to the same minimum and maximum FRET values. The increase in SERCA Ca^2+^ affinity with AMPPCP (Fig. 1*D*, *red arrow*) is consistent with previous studies that show that nucleotide binding allosterically increases SERCA’s affinity to subsequently bind Ca^2+^ (nucleotide activation) (11, 13, 14).

Next, we investigated the effect of co-expression of PLB on Ca^2+^ binding to SERCA. We compared unlabeled WT-PLB to unlabeled S16E-PLB. This mutation mimics phosphorylation by PKA, which relieves inhibition of SERCA by PLB *in vivo* (3, 19, 20, 21) (Fig. S3). In the absence of nucleotide, we did not observe any effect on SERCA Ca^2+^ affinity from co-expression of WT- or S16E-PLB (Fig. 1, *C* and *D* and Table S1). Interestingly, when AMPPCP nucleotide was present, PLB significantly increased the K_Ca_ of SERCA to 702 ± 248 nM (*p* = 0.003) compared to SERCA alone (Fig. 1*D*, *green arrow*). This inhibitory effect of PLB on SERCA Ca^2+^ affinity was prevented by S16E mutation (K_Ca_ = 334 ± 97 nM, *p* = 0.004 compared to WT-PLB) (Table S1). We also evaluated the biosensor response to nucleotide binding. The ATP dependence of the biosensor (K_ATP_ = 10 μM) was compatible with the known ATP-affinity of SERCA (22), and ATP binding was not significantly altered by co-expression of PLB (Fig. S4). Since SERCA ATP-binding affinity was not changed by PLB binding, we conclude the PLB effect on SERCA Ca^2+^ binding affinity (Fig. 1, *C* and *D*) must be due to a disruption of the allosteric effect of ATP on Ca^2+^ binding, rather than an indirect effect, such as loss of allosteric activation through decreased ATP binding.

Repeated measurements of SERCA K_Ca_ in the absence and presence of nucleotide are summarized in Figure 1, *E* and *F*, respectively. Taken together, these results suggest that PLB has no effect on Ca^2+^ affinity in the absence of nucleotide (Fig. 1*E*) in agreement with past results (6, 8, 9) (Fig. 1*F*). However, when a nucleotide is bound to SERCA, the pump binds Ca^2+^ with much higher affinity (11, 12, 13, 14). Under these more physiological conditions, PLB mediates its primary function of inhibiting SERCA Ca^2+^ affinity by reversing allosteric activation of the pump by ATP, shifting the curve partially back to the right (Fig. 1*F*). Others have previously speculated that the presence of nucleotide might be necessary to detect the inhibitory effect of PLB (23, 24), but to our knowledge, this is the first observation that PLB decreases the equilibrium binding affinity of the Ca^2+^ binding sites of non-cycling SERCA. As expected, we observed high Ca^2+^ affinity for SERCA co-expressed with S16E-PLB, consistent with relief of inhibition after PLB phosphorylation by PKA (Fig. 1*F*). The mean values determined for the K_Ca_ and Hill Coefficient are listed in Supplementary Table S1.

### MD simulations of SERCA with ATP and PLB

**Figure 2:**
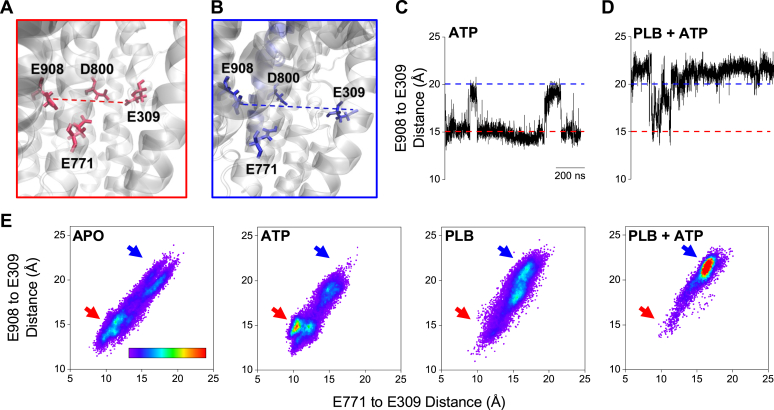
**Effects of ATP and PLB on the structure of SERCA Ca**^**2+**^**transport sites.***A*, arrangement of Ca^2+^-binding residues in a high-affinity configuration, where E309 faces the binding pocket and completes the binding sites. *B*, a low-affinity configuration, where E309 is oriented away from the binding pocket, deforming the binding sites. *C* and *D*, quantification of E908-E309 distance over the MD trajectory revealed rapid interconversion between the high-affinity conformation (short distance, *red dotted lines*) and low-affinity conformation (long distance, *blue dotted lines*). *E*, 2D density maps of the distribution of distances from E309 to E908 and E771. The color scale bar represents the density of points ranging from 0.1% (*purple*) to 21% or more (*red*) occupancy. *Red* and *blue arrows* indicate the E309 position for the high-affinity and low-affinity conformations, respectively. The data suggest the SERCA structure samples the high-affinity conformation more frequently when ATP is present and prefers the low-affinity conformation when PLB and ATP are bound.

To investigate how nucleotide activation and PLB inhibition impact the structure of SERCA, we performed molecular dynamics (MD) simulations of SERCA with and without ATP bound within the N domain and also in the presence and absence of the full-length PLB bound within its regulatory cleft. To evaluate how ATP and PLB impact the structural dynamics of the Ca^2+^ transport sites of SERCA, we analyzed the root mean square fluctuation (RMSF) values for the acidic residues responsible for coordinating Ca^2+^ ions in the binding sites: E309, E771, D800, and E908 (25, 26). We noted that the Ca^2+^ gating residue, E309, was highly dynamic on this microsecond timescale compared to the other residues, as indicated by higher RMSF values across all trajectories, while E908 was stable, with low RMSF (Fig. S5). The Ca^2+^-binding sites showed rapid interconversion between two major geometries: (1) a closed, "high affinity" conformation where E309 faces the binding pocket and completes the binding sites (Fig. 2*A*) and (2) a more open, "low affinity" conformation where E309 is oriented away from the other residues, deforming the Ca^2+^ transport sites (Fig. 2*B*) (26, 27). We monitored the time-dependent change in distance between these residues as an index of the two major geometric conformations, with the closed conformation corresponding to a short distance from E908 to E309 (∼15 Å) (Fig. 2*A*, *red dotted line*) and the open conformation characterized by a long distance (∼20 Å) (Fig. 2*B*, *blue dotted line*). Those distances are also highlighted with horizontal dotted lines in the trajectories shown in Figure 2*C* and *D*. Importantly, transitions between these alternative conformations do not represent the larger, slower conformational changes between E1 and E2 states of the SERCA enzymatic cycle. Rather, the conformations may be considered structural substrates that interconvert on a ns timescale. ATP binding at the remote nucleotide binding sites induced a significant ordering of the Ca^2+^ binding sites, with a more frequent sampling of the short E908-E309 distance compared to APO-SERCA (Fig. 2*C*). (*Additional replicate trajectories are provided in* Supplementary Fig. S6). This stabilization of the Ca^2+^-competent conformation is compatible with the observed ATP-dependent increase in Ca^2+^ affinity (Fig. 1*C*). Interestingly, PLB reversed this nucleotide activation, and the PLB + ATP condition was characterized by a greater sampling of the long E908-E309 distance (Fig. 2*D* and S6). Triangulation of the dynamic E309 residue with E908 and E771 provided additional insight into an apparent disorder-order transition induced by ATP binding. Figure 2*E* provides a 2-dimensional density map of the E309-E908 and E309-E771 distances sampled during the simulation, revealing a wide distribution of distances for the APO condition, with two broad peaks representing the closed (Fig. 2*E*, "APO", *red arrow*) and open conformations (Fig. 2*E*, "APO", *blue arrow*). After the addition of ATP, there was a marked disorder-to-order transition, resulting in a sharply focused peak at short distances (Fig. 2*E*, "ATP", *red arrow*). This signifies a shift to a more stable, well-defined binding pocket, consistent with a high-affinity conformation. This highly ordered nucleotide-activated state of SERCA was abolished by the addition of PLB, resulting in a more frequent sampling of the low-affinity conformation (Fig. 2*E*, "PLB", *blue arrow*). Notably, when PLB and ATP were both bound, the distance distribution showed a sharp, highly populated focus (Fig. 2*E*, "PLB + ATP", *blue arrow*). The build-up of this well-defined peak suggests stabilization of a well-defined inhibitory structure. Since the APO and PLB-bound conditions show the same low Ca^2+^ affinity in the absence of nucleotide (Fig. 1*E*), we conclude that high-affinity Ca^2+^ binding requires the population of an ordered, closed conformation of the binding pocket (Fig. 2*E*, "ATP", *red arrow*). This structure is replaced with a well-defined open structure when PLB binds, reversing nucleotide allosteric activation, and inhibiting SERCA Ca^2+^ affinity.

### Allosteric path analysis

The changes observed in the structure of the Ca^2+^ binding sites with the addition of ATP suggest a long-range allosteric connection between the binding sites for Ca^2+^ and ATP. To explore the allosteric network in the SERCA structure, we analyzed the molecular dynamics trajectories of APO-SERCA, SERCA + ATP, SERCA + PLB, and SERCA + ATP + PLB with GSATools (28, 29). GSATools is an information theory-based software package designed to identify allosteric pathways in protein structures based on the conformational dynamics of local structures and the functional correlations between local and global motions (28, 29). This method has been successfully implemented to predict allosteric paths in diverse proteins (30, 31, 32). Using this framework, the SERCA protein backbone was represented by a sequence of fragments (f) consisting of four residues, with two consecutive fragments sharing three residues. The local structural states of these fragments were assigned conformation states within a structural alphabet based on changes in bond and torsion angles of the fragment backbone over the course of the trajectory (33). These local conformation state changes within fragments were quantified and correlated between all possible pairs of fragments to establish a network that can be analyzed for mutual information (34) within the protein structure. The network constructed for SERCA was analyzed using fragments associated with ATP binding and autophosphorylation sites as a starting point of the pathway. Fragments related to the Ca^2+^ binding sites were used as an endpoint in the pathway. We independently analyzed the trajectories of SERCA for the four conditions described above.

**Figure 3:**
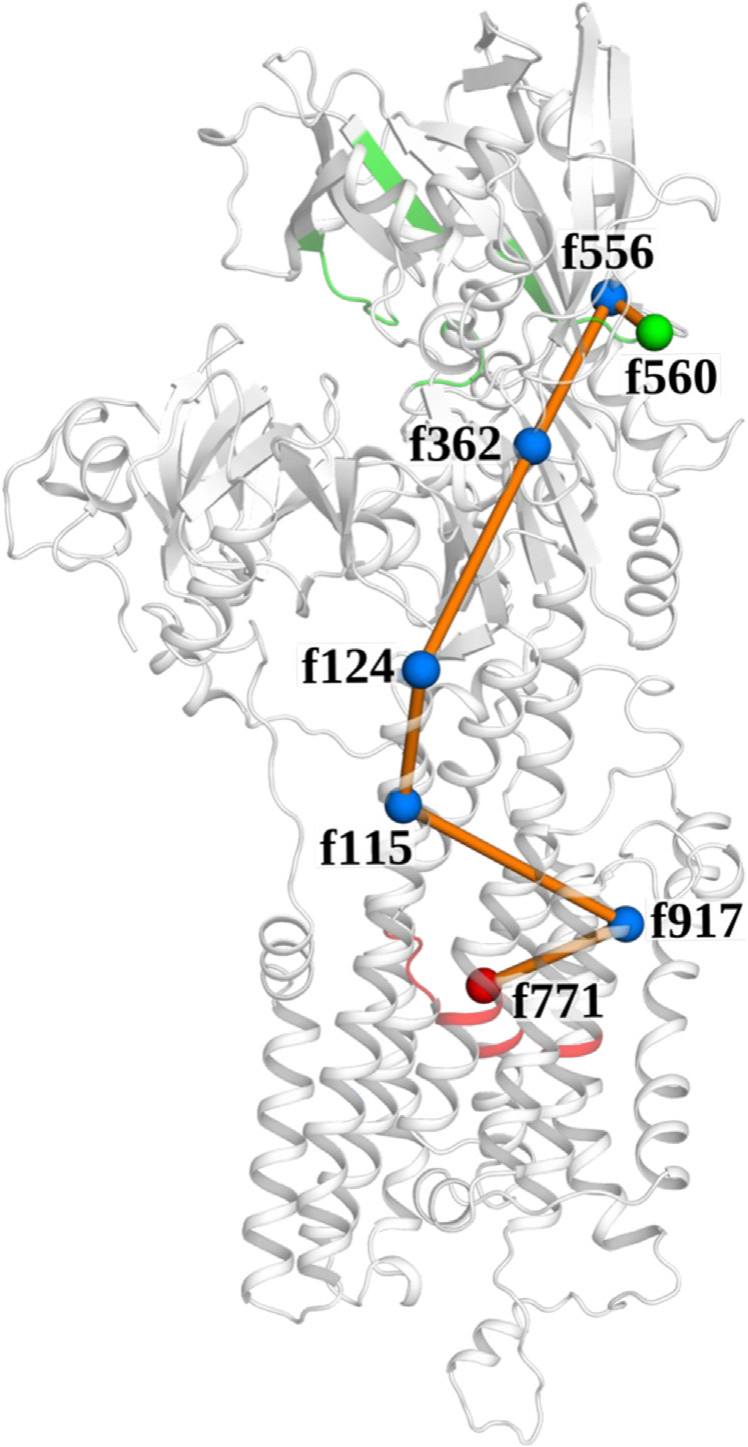
**Mapping the allosteric pathway for nucleotide activation of SERCA.** Analysis of MD simulations of SERCA ± ATP/PLB with GSATools revealed an allosteric pathway (*orange line + blue dots*) that coupled the structural dynamics of the ATP- (*green*) and Ca^2+^-binding sites (*red*) of SERCA in trajectories with ATP bound. PLB binds with residues in fragment 115 (*f115*) in TM2 and disrupted the allosteric coupling of this pathway.

The analysis did not yield an allosteric path for APO-SERCA, which showed only low mutual information connections between fragments. The addition of ATP significantly improved the coupling between the two endpoints, and we obtained an allosteric path passing through the following fragments: f560→f556→f362→f124→f115→f917→f771 (Fig. 3). The residues corresponding to each fragment in this path are shown in Table 1. Of note, the path passed through f115 of TM2, which is part of the PLB regulatory cleft in the transmembrane domain of SERCA. This suggests that PLB could modulate the allosteric signal from the ATP-binding site to the Ca^2+^ binding site by interacting with residues within this fragment. Indeed, we did not detect an allosteric path for the SERCA + ATP + PLB structure trajectories, as the Ca^2+^ and ATP binding sites were either not connected in the network or connected with edges having low mutual information. We conclude that PLB binding to SERCA inhibits the allosteric communication between ATP and Ca^2+^-binding sites and reverses nucleotide activation.

**Table 1:** Residues identified in the allosteric path coupling the ATP and Ca^2+^ binding sites

Fragment name	Residues
f115	Ala115, Glu 116, Asn117, Ala118
f124	Glu124, Tyr125, Glu126, Pro127
f362	Asn362, Gln363, Met364, Ser365
f556	Gly556, Thr557, Gly558, Arg559
f560	Asp560, Thr561, Leu562, Arg563
f771	Asn771, Val772, Gly773, Glu774
f917	Asn917, Ser918, Leu919, Ser920

## Discussion

In previous studies, we (35, 36, 37) and others (6, 24, 38, 39) have investigated SERCA regulation by PLB under the premise that PLB slowed the kinetics of the structural transition associated with Ca^2+^ binding to SERCA, altering the pump's "apparent" Ca^2+^ affinity without changing the actual affinity of the Ca^2+^-binding sites. This perspective was based on Ca^+2^-binding experiments performed in the absence of ATP (6, 8, 9), a condition routinely used to stop SERCA enzymatic cycling for equilibrium measurements. In those studies, investigators observed no effect of PLB on Ca^2+^ binding at equilibrium and concluded that there is no direct effect of PLB on "actual" SERCA Ca^2+^ affinity (6). The inhibitory effect of PLB was attributed to kinetics, as PLB was hypothesized to slow the transition between E2 and E1 (24). Hence, the terminology "*apparent affinity*" has long been used in the field, specifically to discount the effect of PLB on equilibrium Ca^2+^ affinity.

In the present study, we prevented enzymatic cycling of the pump using a non-hydrolyzable analog of ATP, which enabled quantification of Ca^2+-^binding to the nucleotide-bound SERCA under equilibrium conditions. These experiments also exploited a fluorescent biosensor, “2-color SERCA”, that reports the Ca^2+^-dependent conformation change in the SERCA headpiece with a change in FRET (15). Although this is an indirect measure of Ca^2+^ binding, this assay offers an advantage of 2 to 3 orders of magnitude improved sensitivity compared to conventional quantification of ^45^Ca^2+^ binding to cardiac SR (6). Using this alternative approach, we obtained results that recapitulate several key observations from previous studies. We found that the presence of nucleotide greatly increased the Ca^2+^ sensitivity of SERCA (Fig. 1*F*), in agreement with others (10, 11, 12, 13, 14). This phenomenon is referred to as “nucleotide activation.” We also reproduced the observation that there is no effect of PLB on equilibrium Ca^2+^ binding to SERCA in the absence of nucleotide (Fig. 1*E*) (6, 8, 9). However, we find PLB *does* decrease SERCA’s equilibrium Ca^2+^ binding in the presence of non-hydrolyzable nucleotide (Fig. 1*F*). Indeed, the twofold change in the K_Ca_ of the FRET response with PLB is the same as the 2-fold change in the K_Ca_ of the ATPase activities previously reported by others (40, 41, 42, 43), so the observed change in Ca^2+^ binding affinity can fully account for the effect of PLB on the cycling pump. Our interpretation of these results represents a departure from the previous model. Rather than PLB having a purely kinetic effect, we find that PLB alters the actual equilibrium Ca^2+^ binding affinity of SERCA. Importantly, the data are still compatible with the observation that PLB slows the structure transitions associated with Ca^2+^ binding, but we suggest that the cause-effect relationship is inverted compared to the previous paradigm. Rather than a slower structural transition causing a change in SERCA's apparent Ca^2+^ affinity, we propose that the decrease in SERCA Ca^2+^ affinity causes the structural transition associated with Ca^2+^ binding to be slower.

MD simulations provided insight into the mechanism by which PLB modulates allosteric communication between the ATP- and Ca^2+^-binding sites. Of particular interest were the dynamic motions we observed in glutamine 309 (Fig. 2, *A* and *B*). This residue has well-established roles in both gating the entry of Ca^2+^ ions into the binding cavity (26, 27) and allosterically coupling the structure of the TM and headpiece domains of SERCA (44, 45, 46). In the APO condition, we observed this residue moving in and out of the Ca^2+^ binding pocket, consistent with crystal structures that have shown E309 in either position. E309 faces the binding pocket in Ca^2+^-bound states of SERCA (47, 48) but faces away when thapsigargin is bound and inhibits Ca^2+^ binding (26). ATP and PLB both impacted the Ca^2+^ binding sites by affecting the equilibrium position of this residue. In simulations with ATP bound, our results showed E309 more frequently occupied a well-defined position in the closed conformation (Fig. 2*D*, "ATP"), suggesting that ATP binding to SERCA allosterically primes the transporter for Ca^2+^ binding. This agrees with past biochemical observations that E309 is important for mediating the nucleotide-dependent enhancement of SERCA Ca^2+^ binding and occlusion (27). Interestingly, simulations with PLB bound to SERCA showed the opposite trend, with the PLB + ATP condition exhibiting a strong preference for occupying the more open state of E309 (Fig. 2*D*, "PLB + ATP"), where the Ca^2+^ binding site is distorted (Fig. 2*B*). This suggests that the PLB interaction with SERCA disrupts the allosteric communication between the ATP and Ca^2+^ binding sites and stabilizes the pump in a conformation that binds Ca^2+^ poorly.

The allosteric network analysis provided additional insight into the results of the biochemical measurements and MD simulations. This analysis revealed a novel allosteric pathway that couples ATP- and Ca^2+^-binding sites of SERCA when ATP is bound (Fig. 3). Interestingly, this route passes through the PLB binding cleft residues on TM2. The presence of PLB disrupts the structural coupling between the remote ligand-binding sites, most likely by altering the conformations of the residues in the PLB-binding regulatory site: A115, E116, N117, and A118. It was noteworthy that E771 was the end point of the predicted pathway because we have previously shown that this residue plays a key role in the PLB-mediated modulation of SERCA (36). The increased FRET in response to nucleotide at low Ca^2+^ concentration (Fig. 1*C*) suggests that ATP binding still causes headpiece closure when PLB is present. However, this first step in the ATP activation mechanism is not transmitted to the Ca^2+^-binding sites when PLB disrupts the allosteric pathway, uncoupling the transmembrane domain from conformational changes in the headpiece. Since this allosteric pathway is utilized by both nucleotide activation and PLB inhibition of the pump, the structural elements along this pathway may present new targets for pharmacological stimulation or inhibition of SERCA.

It is a limitation of this analysis that the PLB-SERCA crystal structure does not reveal the PLB cytoplasmic domain, so the PLB cytoplasmic domain was modeled on the NMR structure solution (36, 49, 50) without imposing specific interactions of that domain with the SERCA cytoplasmic headpiece. Thus, the results do not shed light on other functionally important connections between the PLB cytoplasmic domain IA and the SERCA P-domain, and we cannot determine how PLB phosphorylation is allosterically communicated through the PLB and/or SERCA structures to relieve inhibition of Ca^2+^-binding sites. Future structure solutions and computational studies may offer new opportunities to extend this analysis to that mechanism and could examine PLB domain IB interactions with SERCA cytoplasmic loop M6-M7 (51).

**Figure 4:**
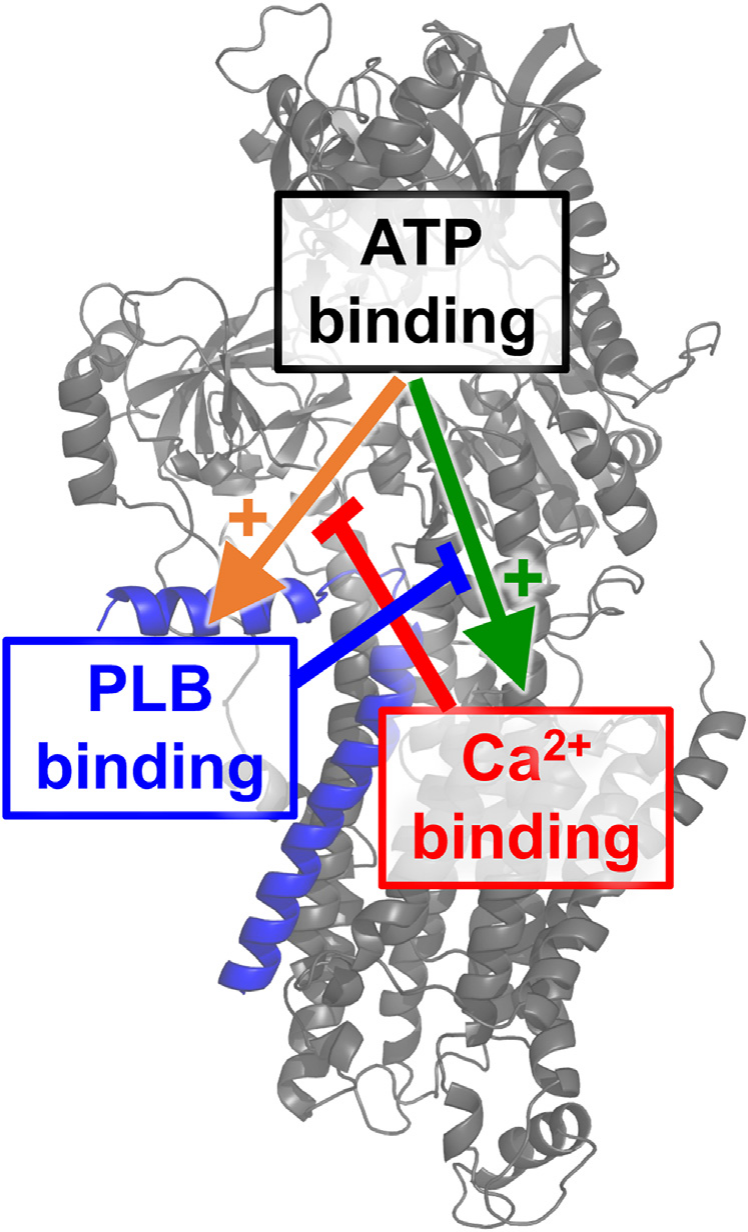
**A schematic diagram of the allosteric interplay between ATP, PLB (*blue*), and Ca**^**2+**^**binding sites on SERCA (*gray*).**

Finally, additional context for these results is provided by our previous study (35), in which we showed that the PLB-SERCA regulatory complex is most stable when the Ca^2+^ pump is in its ATP-bound conformation (24, 52, 53, 54). This ATP-dependent increase in PLB-SERCA affinity was reversed upon Ca^2+^ binding (35). The present study reveals that ATP and PLB may regulate SERCA Ca^2+^ affinity by interacting with a common allosteric pathway. Figure 4 summarizes the allosteric relationships between SERCA binding sites for ATP, Ca^2+^, and PLB. ATP binding to its active site in the N-domain allosterically activates the Ca^2+^ binding sites in the transmembrane domain, increasing SERCA Ca^2+^ affinity (Fig. 4, *green*). We previously showed ATP binding also causes PLB to interact more avidly with SERCA (Fig. 4, *orange*). Here, we demonstrated that PLB inhibits SERCA by disrupting the allosteric coupling of the ATP- and Ca^2+^-binding sites (Fig. 4, *blue*), reversing nucleotide activation of SERCA Ca^2+^ binding. Counterregulation by Ca^2+^ occurs as the affinity of the PLB-SERCA regulatory complex with ATP is reduced after Ca^2+^ is bound (Fig. 4, *red*). Overall, the results provide new insight into a novel allosteric pathway connecting the ATP binding site with distant Ca^2+^ binding sites and reveal that PLB disrupts this path.

## Experimental procedures

### Plasmid constructs

The engineering and functional characterization of our canine 2-color SERCA2a has been previously described (15, 16, 55, 56). Here, we used a version of this construct labeled with mMaroon1 on the N-terminus (labeling the A domain) and a mCyRFP1 intersequence tag inserted before residue 509 on the N domain of SERCA (17). The mCyRFP1 and mMaroon1 donor/acceptor FRET pair has a Förster distance (*R*_*0*_) of 63.34 Å. Our lab has previously demonstrated that the fusion of one or two fluorescent proteins to SERCA did not alter normal Ca^2+^ transport function (15, 16, 56). Furthermore, PLB fused to another tag was able to normally regulate SERA function (15). Therefore, we believe fluorescent proteins are benign for the normal function of SERCA or PLB.

### Molecular biology and cell culture

HEK-293T cells were cultured in Dulbecco’s modified Eagle’s medium (DMEM) supplemented with 10% fetal bovine serum (ThermoScientific). Cells were cultured on 150 mm^2^ dishes and transiently transfected using the Lipofectamine 3000 transfection kit (Invitrogen) with either (1) 90 μg of 2-color SERCA plasmid DNA alone or (2) 50 μg of 2-color SERCA plasmid DNA and unlabeled PLB plasmid DNA supplemented at a 1:3 or 1:5 SERCA to PLB ratio (150 or 250 μg unlabeled PLB, respectively). Cell lines were tested every 6 months to ensure they were free of *mycoplasma*.

### HEK-293T cell microsomal membrane preparation

ER microsomal membranes expressing 2-color SERCA were isolated as previously described (16). Briefly, roughly 48 h post-transfection, cells expressing 2-color SERCA were washed with PBS, harvested by scraping in 20 ml of an ice-cold homogenizing solution containing 10 mM Tris-HCl pH 7.5, 0.5 mM MgCl_2_, and an EDTA free protease inhibitor cocktail, and pelleted by centrifugation at 1000*g* for 10 min at 4 °C. Cell pellets were resuspended in 5 ml of cold homogenizing solution and disrupted by 10 strokes in a Potter-Elvehjem homogenizer. Cell homogenates were then supplemented with 5 ml of ice-cold sucrose solution (100 mM MOPS pH 7.0, 500 mM sucrose, and an EDTA-free protease inhibitor cocktail) and passed through a 27-gauge needle 10 times. Cell homogenates were then centrifuged at 1000*g* for 10 min at 4 °C. The supernatants were collected and centrifuged at 126,000*g* for 30 min at 4 °C. High-speed membrane pellets were resuspended in a 1:1 mixture of homogenizing and sucrose solutions, disrupted by 10 strokes in a Potter-Elvehjem homogenizer, and passed through a 27-gauge needle 10 times. A Pierce BCA assay kit (ThermoScientific) was used to determine the protein concentration of membrane preparations.

### Time-correlated single-photon counting

Fluorescence lifetime measurements were obtained from microsomal membrane preparations from HEK-293T cells expressing 2-color SERCA labeled with mMaroon1-and mCyRFP1- labeled on the A and N domains respectively, with or without unlabeled PLB co-expressed. Membranes were diluted at a 1:10 ratio in a solution containing 100 mM KCl, 5 mM MgCl_2_, 10 mM imidazole, 2 mM EGTA, and varying concentrations of CaCl_2_. For the nucleotide-bound condition, 500 μM AMPPCP was added to the solution. This non-hydrolyzable nucleotide was used in place of ATP for all experiments except when measuring the dose-response of 2-color SERCA to ATP (Fig. S4). Independent experiments were performed in the presence and absence of AMPPCP using seven microsomal preparations expressing 2-color SERCA alone and six preparations where WT/S16E-PLB was co-expressed. The mCyRFP1 donor was excited with a supercontinuum laser (FIANIUM) filtered through a 482/18 nm bandpass filter. Emitted fluorescence from the sample was detected through a 1.2 N A. water immersion objective and transmitted through a 593/40 nm bandpass filter to a PMA Hybrid series detector (PicoQuant). Light from the detector was quantified by a HydraHarp 400 single-photon counting module at 16 ps resolution. TCSPC histograms were obtained over 60 s acquisition period for each condition. In control experiments with singly-labeled mCyRFP1-SERCA, the donor alone gave a single-exponential decay with a *τ*_*D*_ of 3.52 ns. TCSPC histograms from 2-color SERCA samples were fit with a two-exponential decay function, which was used to derive the amplitude-weighted average lifetime for the two 2-color SERCA populations (*τ*_*DA*_). FRET efficiencies were calculated for each sample according to the relationship $FRET=100*\left(1-\frac{\tau_{DA}}{\tau_{D}}\right)$ (57) and plotted as a function of Ca^2+^ concentration. The data were well described by a Hill function of the form $y=START+\frac{\left(END-START\right)*x^{n}}{K^{n}+x^{n}}$, where *START* is the minimal FRET efficiency at low Ca^2+^, *END* is the maximum FRET efficiency at high Ca^2+^, *n* is the Hill coefficient, and *K* is the Ca^2+^ binding constant (K_Ca_). Data from eight independent experiments from a minimum of four microsomal preparations were global fit to obtain a single best Hill coefficient with independent K_Ca_ values for each experimental condition. Differences in K_Ca_ were evaluated by one-way ANOVA with Dunn’s Sidak *post-hoc* test. For Ca^2+^ binding measurements with microsomes co-expressing 2-color SERCA and unlabeled WT-PLB, we evaluated 3:1 and 5:1 PLB to SERCA expression ratios. We did not detect a significant difference in Ca^2+^ affinity between these samples (58).

### Preparation of the systems

We used the crystal structure of SERCA (PDB 3w5a) to simulate the SERCA apo and SERCA–ATP complexes. At physiological pH SERCA populates mainly the E1 state, even in the absence of Ca^2+^ (59, 60), so we considered the Mg^2+^-bound structures of SERCA in the E1 state to be the appropriate starting point to simulate apo SERCA. To simulate the SERCA–PLB systems, we used the atomic model of the full-length complex previously reported by us (61). To model the ATP-bound structure, we docked ATP onto the nucleotide-binding pocket of SERCA using the CB-DOCK program (62). K^+^ was used in place of Ca^2+^ as previously described (63). Others have shown that the K^+^-bound state is a necessary step for Ca^2+^ binding to SERCA (64). Indeed, increasing the K^+^ concentration from 0 to 100 mM produces a 4-fold increase of the rate constant of the Ca^2+^-induced fluorescence change and an 8-fold increase of the rate constant of the EGTA-induced fluorescence change, and rapid filtration assays showed that K^+^ binding increases the rate of ^45^Ca^2+^-^40^Ca^2+^ exchange reaction (64). Molecular simulations also showed that K^+^ binding to the Ca^2+^ sites is a step necessary to produce a competent transport site geometry that is capable of recognizing and binding Ca^2+^ (63). In our previous meta-analysis of all crystal structures of SERCA reported in the literature we used root mean square deviation (RMSD) to quantitatively compare and cluster together structures that represent the structural states populated along the catalytic cycle of the pump (2). This analysis showed that the crystal structures of APO or ATP-bound states are structurally identical to those of the PLB-bound pump and that both structures represent the E1 state of the pump. We adjusted the pK_a_ of other ionizable residues to a pH value of ∼7.2 using PROPKA (65, 66). The complexes were embedded in a 120 × 120 Å bilayer of POPC lipids. The initial system was solvated using TIP3P water molecules with a margin of 20 Å in the z-axis between the edges of the periodic box and the cytosolic and luminal domains of SERCA, respectively. K^+^, and Cl^−^ ions were added to neutralize the system and to produce a KCl concentration of ∼100 mM. Preparation of the systems was done using the CHARMM-GUI web interface (67).

### Molecular dynamics simulations

We performed molecular simulations with AMBER20 on Tesla V100 GPUs (68) using the AMBER ff19SB force field (69). We maintained a temperature of 310 K with a Langevin thermostat and a pressure of 1.0 bar with the Monte Carlo barostat. We used the SHAKE algorithm to constrain all bonds involving hydrogens and allow a time step of 2 fs. We first performed 5000 steps of steepest-descent energy minimization followed by equilibration using two 25-ps MD simulations using a canonical ensemble (NVT), one 25-ps MD simulation using an isothermal–isobaric ensemble (NPT), and two 500-ps MD simulations using the NPT ensemble. The equilibrated systems were used as a starting point to perform the production MD simulations.

### Information-theoretic analysis of allosteric paths in SERCA

We analyzed MD simulations from four configurations of SERCA: APO-SERCA, SERCA + ATP, SERCA + PLB, and SERCA + ATP + PLB. The trajectories for the four configurations were analyzed to detect the allosteric path between ATP binding/autophosphorylation site and Ca^2+^ binding site using GSATools to determine conformational dynamics and functional correlations between local and global motions (28, 29). GSATools is based on analyzing the correlations in the local dynamics of the protein backbone. The local motions are important in allostery signal propagation as many residues, which are involved in conformational changes also have significant changes in the dihedral angles of the protein backbone (70). Other components of protein dynamics, such as the motion of side chains and the collective motion of protein, are taken into account indirectly through their effect on the local motions of the protein backbone. The protein backbone was represented by overlapping fragments consisting of four residues. At each point in the trajectory, the fragments are assigned a conformational state, which uniquely depends on a set of three independent internal angles between the Cα atoms of the fragment (33). For each fragment, two of these internal angles are the pseudo bond angles between the 4 Cα atoms in the fragment, and the third angle is the torsion angle between the Cα atoms in the fragment. Based on the values of these angles, the local structural states of these fragments throughout the trajectory were determined using a structural alphabet, as previously described (33). The structural alphabet describes the most likely local conformations for a protein fragment from a defined set of 25 canonical states, and each fragment is assigned a most similar structural alphabet letter (A-Y) depending on the RMSD values using local fit approximation (71). Hence, a protein with n residues is represented as a structural string of length n − 3. The conformational state of a protein can thus be encoded into a structural string, which is a sequence of alphabets representing the local states of each fragment. In this way, we obtain a set of aligned structural strings, a column that describes all the possible conformational states sampled by a fragment throughout the simulation trajectory. Next, we discuss the model for signal propagation based on the correlated changes in fragment states. The propagation of an allosteric signal is modeled as information exchange in a network based on the correlation in the local motions of the protein (28, 29). The local motions are taken into account as transitions between the canonical states of the fragments, and the coupling of these fragment transitions is time-averaged over the whole trajectory. The correlation of the transitions in the conformational states of a fragment pair is determined by the mutual information (MI). The MI between fragments was calculated to determine the correlation between the local states of the fragments. The normalized mutual information between two fragments *i* and *j*, is represented by $I_{LL}^{n}$ is,

$$I_{LL}^{n}\left(C_{i};C_{j}\right)=\frac{I\left(C_{i};C_{j}\right)\;-\;\epsilon \left(C_{i};C_{j}\right)}{H\left(C_{i};C_{j}\right)}$$

Here, *C*_*i*_ and *C*_*j*_: columns *i* and *j* in the string alignment. *I(C*_*i*_*; C*_*j*_*)*: mutual information, *H(C*_*i*_*, C*_*j*_*)*: joint entropy of two fragments. *ε(C*_*i*_*; C*_*j*_*)*: error term arising from error due to finite size of data and quantization of continuous random variables (72). A pairwise matrix of $I_{LL}^{n}$ for all fragment pairs is calculated. These interactions between the fragments (quantified by $I_{LL}^{n}$ values) are modeled by an undirected weighted network, with each node in the network representing a protein fragment. The nodes of the network are connected by the edges where the MI between the two fragments determines the weight of the edge between a node pair (i, j) and is given by the relation,

$$w_{ij}=1\;-\;I_{LL}^{n}\left(C_{i};C_{j}\right)$$

So, a fragment pair with a high value of $I_{LL}^{n}$ (high information exchange) is represented by a node pair connected by an edge with low weight in the network. In this analysis, an appropriate value of the distance cutoff and MI cutoff must be assigned. The distance cutoff sets the maximum physical distance between the first Cα atoms of the fragment pair for which an edge connection between the fragments can exist. A relevant value of cutoff for the MI between the nodes $I_{LL}^{n}$ have also to be set so that the edges with high weights (or low $I_{LL}^{n}$) can be neglected. The network thus constructed for a protein is sensitive to these cutoffs. In our analysis, we set the distance cutoff to 30 Å and the $I_{LL}^{n}$ the cutoff was taken to be 33% of the maximum value of MI between any pairs of fragments for the four configurations and is equal to 0.133. So, a pair of nodes will have an edge if the $I_{LL}^{n}$ is in the top 67% of all the $I_{LL}^{n}$ between different pairs of fragments and within the distance cutoff of 30 Å. Ideally, one should take the MI between the fragments $I_{LL}^{n}$ to be greater than 50%. We set a lower cutoff as we did not get a path between two binding sites for a greater cutoff (with a distance cutoff of 30 Å) for SERCA-ATP. Additionally, some of the fragments in the predicted allosteric path can be distant from each other in physical space (although within the distance cutoff). These fragment pairs must have intermediate fragments between them as part of the allosteric pathway, but these fragments are not uniquely discernable from the analysis of the simulated trajectories.

The endpoints of the possible allosteric network are taken as the fragments associated with Site A (ATP binding site and auto-phosphorylation site) and Site B (Ca^2+^ binding site.) Since multiple fragments are associated with both Site A and Site B, all the possible combinations of fragments from the two sites were considered for the analysis. For all four cases, we first ascertain whether the paths exist between sites A and B and then determine the shortest paths using Dijkstra’s algorithm (73). The shortest path is the path with the lowest total weight among all possible paths between Site A and Site B in the network and, hence, is deemed as the most optimal path for allosteric signal propagation between the two sites. A relevant quantity of interest here is the node eigenvector centrality score, which measures the relative importance of each node in the network (73). The highest-centrality nodes, which have a large number of connections and are likely to be involved in allosteric signal transmission, were determined for each case. Figure S7A depicts the high centrality nodes for the SERCA + ATP trajectory. All the fragments in the predicted allosteric path except the endpoint fragments lie in the range of the top 12% of the eigenvector centrality values among all fragments. To identify the fragments that are likely to be associated with the collective global motion of the protein and hence are important in determining the allosteric path (28, 29), a normalized mutual information $I_{\mathrm{LG}}^{n}$ between the local states of the fragment and the global collective motion states of the protein is also calculated for all fragments (see Fig. S7C). A detailed discussion about the method to compute $I_{\mathrm{LG}}^{n}$ is given in Ref. (28).

A strength of the allosteric pathway analysis method is that it does not rely on the simulation timescales. In the GSA analysis, a network is constructed by computing the correlation of the local states of the protein. The correlated transition in states between the local fragments can occur in a timescale that is far less than the total time involved in the allosteric signal propagation. By averaging these changes over the whole trajectory between different local fragments, we identify the fragment pairs that are highly coupled (have high mutual information) and generate a network. An allosteric pathway is then determined between the two endpoints on the network. Importantly, the allosteric pathway analysis does not predict conformational changes but predicts protein residues that are interconnected by an allosteric network. In this way, the analysis predicts that changes in one part of the network can affect the dynamics and structure of another part of the network. Timescales are irrelevant to this analysis, as long as trajectories are sampled adequately in any one given state.

## Data availability

Data are available upon request: Seth L. Robia, srobia@luc.edu.

## Supporting information

This article contains supporting information.

## Conflict of interest

The authors declare that they have no known competing financial interests or personal relationships that could have appeared to influence the work reported in this paper.
