# Supplementary material for: Phospholamban inhibits the cardiac calcium pump by interrupting an allosteric activation pathway
Source: J Biol Chem. 2024 Apr 6;300(5):107267. doi: 10.1016/j.jbc.2024.107267 (PMC11098958; doi:10.1016/j.jbc.2024.107267)
Supplement: Supporting information [file mmc1.pdf]

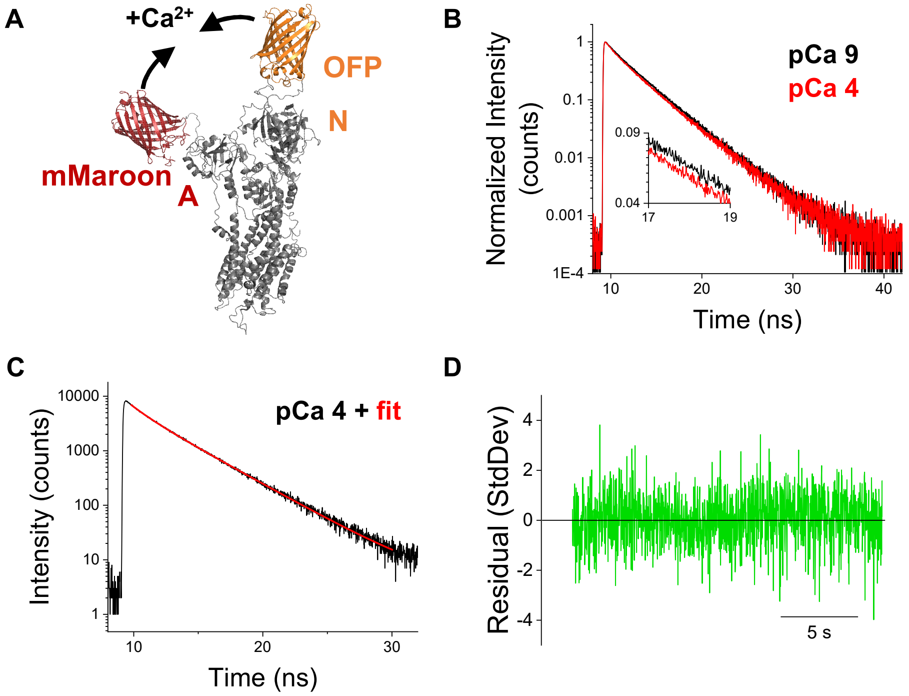

Supplementary Figure S1. A) 2-color SERCA biosensor labeled on the A and N domains undergoes headpiece closure during Ca^{2+} binding by intramolecular FRET. **B)** The fluorescence lifetime of the OFP donor in 2-color SERCA is quenched by the mMaroon acceptor when Ca^{2+} is increased from pCa 9 to pCa 4, due closure of the labeled headpieces (as in panel A). The inset data are zoomed in two make the difference more appreciable. **D)** A representative fit (*red*) of the OFP donor lifetime (*black*) using a two exponential decay function. **E)** A residual of a two-exponential decay fit of the OFP donor lifetime of 2-color SERCA shows that the data are well described by this two species model.

Apparent K_{Ca} in the Absence of Nucleotide (Mean ± SEM)	
Condition	K_{Ca} (μM)
SERCA alone	1.77 ± 0.11
PLB	1.68 ± 0.16
S16E	1.84 ± 0.20

Supplementary Table S1. Apparent Ca^{2+} binding constants derived from intramolecular FRET measurements of 2-color SERCA alone and with WT- or S16E-PLB in the absence of nucleotide.

Apparent K_{Ca} in the Absence of Nucleotide *p* values from 1-way ANOVA with Tukey's post-hoc		
	SERCA alone	PLB
S16E	0.959	0.762
PLB	0.903	

Supplementary Table S2. *P* values comparing differences in apparent Ca^{2+} binding constants of SERCA alone and with WT- or S16E-PLB in the absence of nucleotide. These values were determined by one-way ANOVA with Tukey's *post-hoc* test (* = p<0.05).

Apparent K_{Ca} +Nucleotide (Mean ± SEM)	
Condition	K_{Ca} (μM)
SERCA alone	0.33 ± 0.03
PLB	0.78 ± 0.14
S16E	0.34 ± 0.04

Supplementary Table S3. Apparent Ca^{2+} binding constants derived from intramolecular FRET measurements of 2-color SERCA alone and with WT- or S16E-PLB in the presence of AMPPCP.

Apparent K_{Ca} +Nucleotide *p* values from 1-way ANOVA with Tukey's post-hoc		
	SERCA alone	PLB
S16E	0.999	**0.001***
PLB	**9.78 x 10^{-4}***	

Supplementary Table S4. *P* values comparing differences in apparent Ca^{2+} binding constants of SERCA alone and with WT- or S16E-PLB in the presence of AMPPCP. These values were determined by one-way ANOVA with Tukey's *post-hoc* test (* = p<0.05).

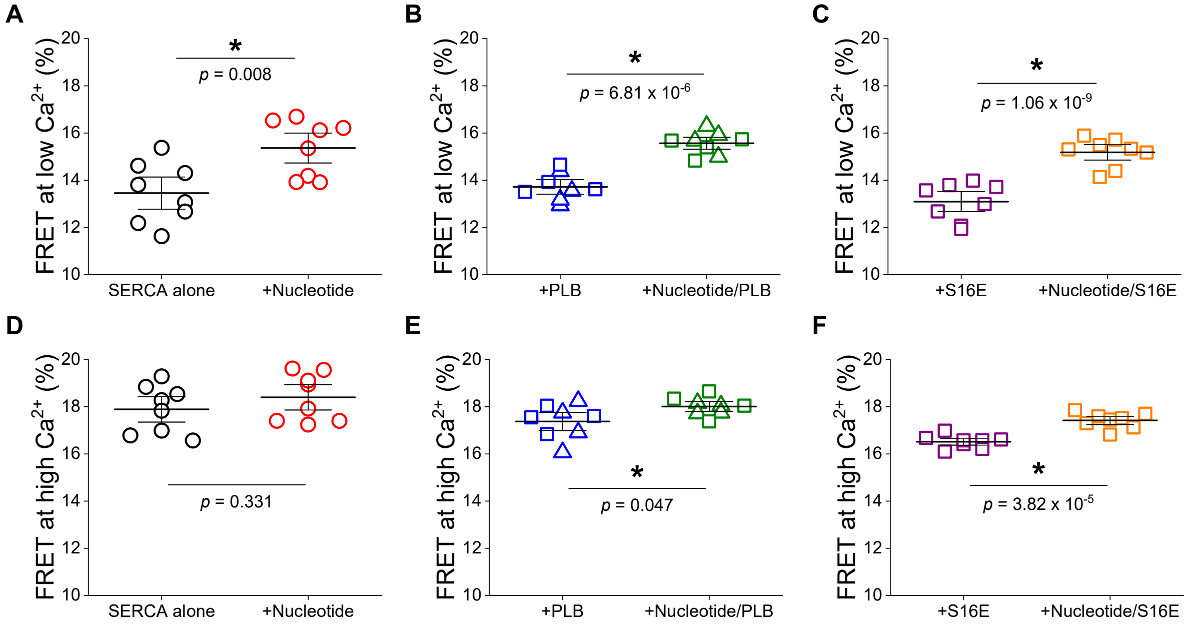

Supplementary Figure S2. The FRET efficiency of 2-color SERCA under low and high Ca^{2+} conditions as determined by fitting the average FRET of the biosensor in a range of Ca^{2+} concentrations with a Hill equation. **A-C)** FRET efficiency of 2-color SERCA at low Ca^{2+} was significantly increased by nucleotide activation in all samples. **D-F)** FRET efficiency of 2-color SERCA at high Ca^{2+} was significantly increased only in samples coexpressing WT- or S16E-PLB. Differences determined by student's t-test (*= p<0.05)

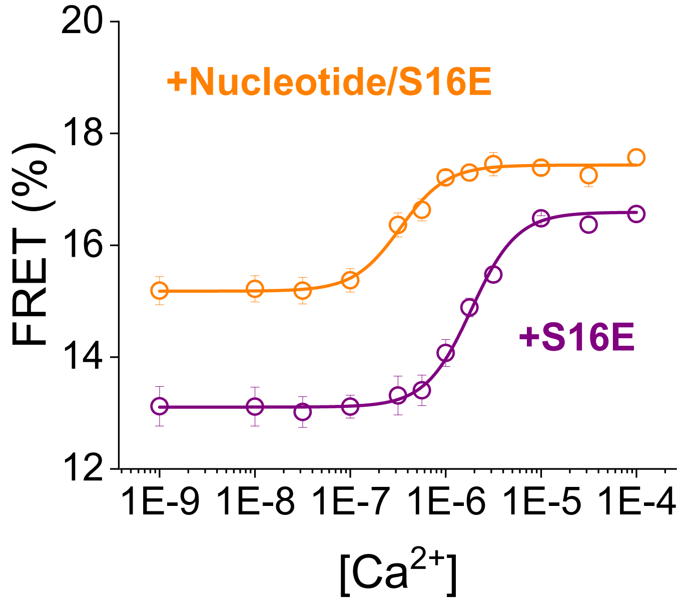

Supplementary Figure S3. 2-color SERCA FRET changes during Ca^{2+} binding measured in samples coexpressing SERCA and S16E-PLB (1:5 SERCA to PLB ratio) in the presence (*orange*) and absence of AMPPCP (*purple*).

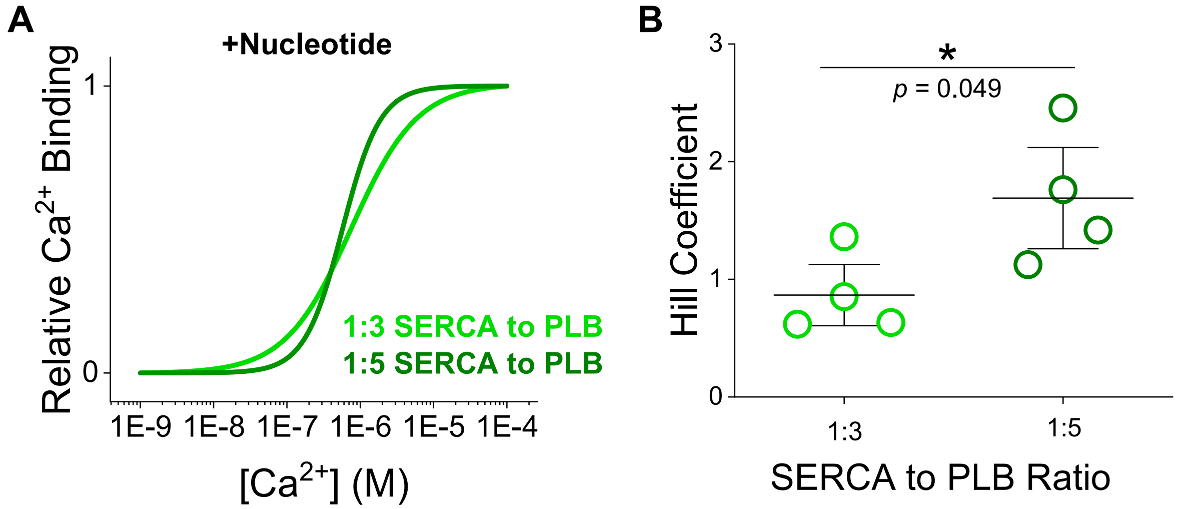

Supplementary Figure S4. A) Normalized fitted curves for 2-color SERCA FRET changes during Ca^{2+} binding in the presence of AMPPCP for samples coexpressing WT-PLB at a 1:3 (*light green*) or a 1:5 (*dark green*) SERCA to PLB ratio. Data are based on fitting the mean and SE of 4 independent measurements. **B)** Hill coefficient values for changes in 2-color SERCA FRET during Ca^{2+} binding for samples coexpressing WT-PLB at a 1:3 (*light green*) or a 1:5 (*dark green*) SERCA to PLB ratio. Hill coefficient values were determined by global fitting the data from 4 independent experiments to share a common K_{Ca} value. Differences determined by student's t-test (*= p<0.05).

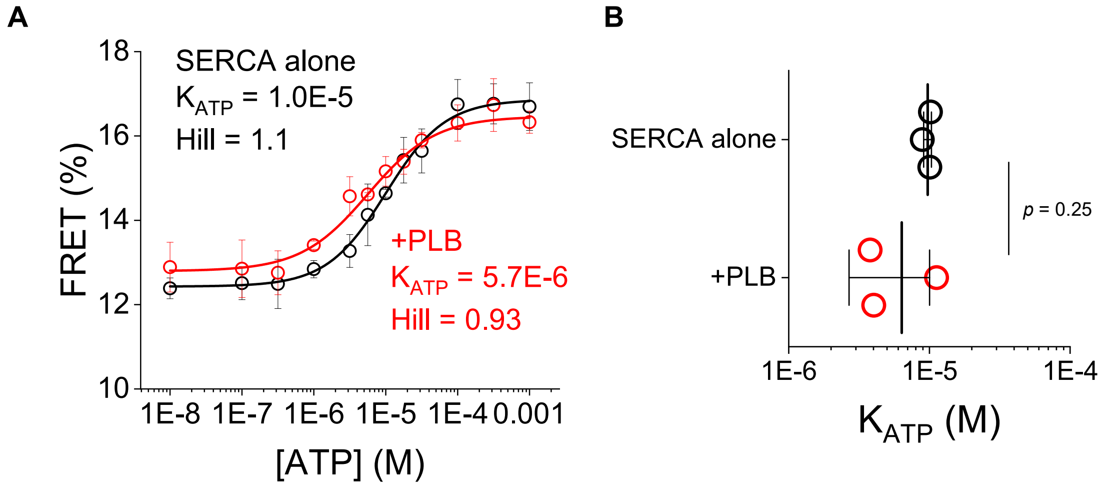

Supplementary Figure S5. A) 2-color SERCA FRET changes during ATP binding measured in samples expressing SERCA alone (*black*) or coexpressing WT-PLB at a 1:5 SERCA to PLB ratio (*red*). **B)** Values for the apparent ATP binding constant (K_{ATP}) of 2-color SERCA determined from 3 independent experiments. Differences determined by student's t-test (*= p<0.05).

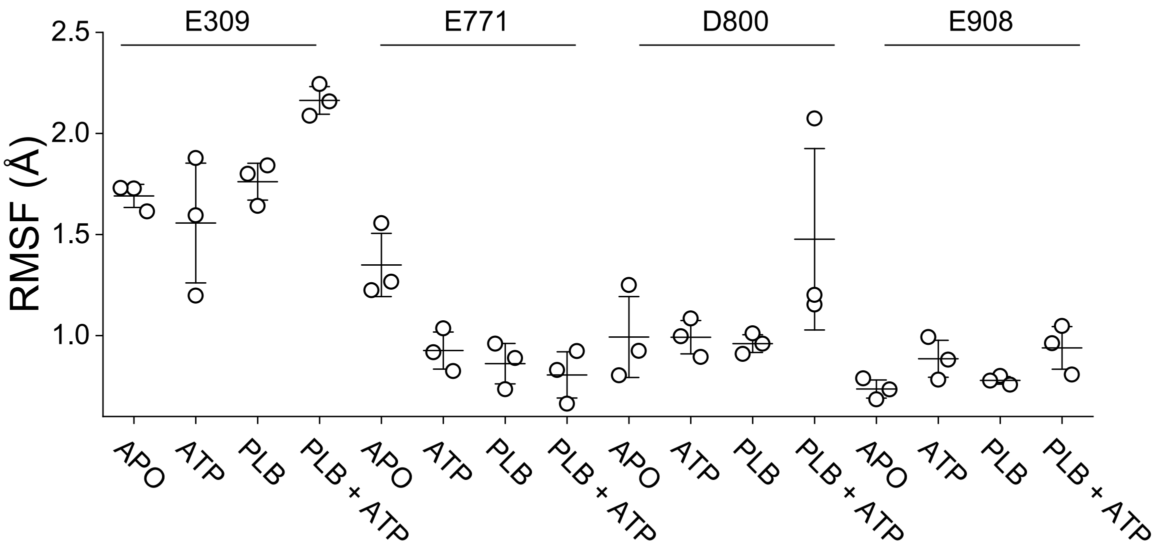

Supplementary Figure S6. RMSF values for the acidic residues that coordinate Ca^{2+} ions in Ca^{2+} transport sites of SERCA.

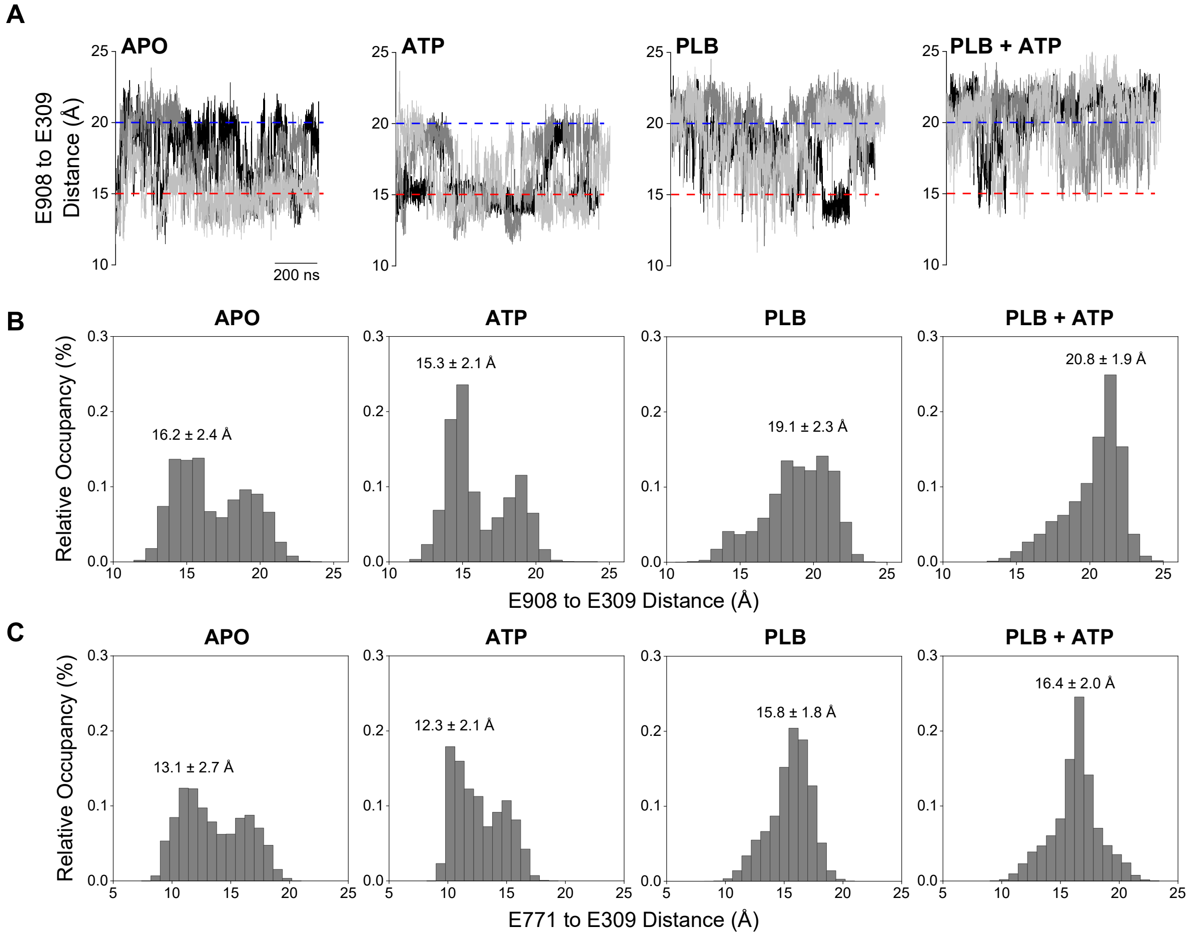

Supplementary Figure S7. A) Time-dependent changes in the distance between E908 and E309 for replicates 1 (*black*), 2 (*grey*), and 3 (*light grey*) for each of the simulated conditions. Red and blue dashed lines indicate the distances of the high and low Ca^{2+} affinity conformations of the binding site represented in Fig. 2B and C respectively. **B)** Relative occupancy of the distances between SERCA Ca^{2+} binding residues E908 and E309 based on 3 independent trajectories. **C)** Relative occupancy of the distances between SERCA Ca^{2+} binding residues E771 and E309 based on 3 independent trajectories. Overlayed values are the median and standard error for each data set.
